# Rheological Properties of Selected Copper Alloys in Liquid and Semi-Solid State

**DOI:** 10.3390/ma19214747

**Published:** 2026-11-08

**Authors:** Arkadiusz Madetko, Platon Kapranos, Krzysztof Sołek

**Affiliations:** 1 Faculty of Metals Engineering and Industrial Computer Science, AGH University of Krakow, al. A. Mickiewicza 30, 30-059 Krakow, Poland; solek@agh.edu.pl; 2 Department of Materials Science & Engineering, Sir Robert Hadfield Building, The University of Sheffield, Mappin Street, Sheffield S1 3J, UK; platonkapranos12@gmail.com

**Keywords:** copper alloys, rheological properties, rheological models, viscosity, thixoforming processes

## Abstract

**Highlights:**

- Viscosity analysis of copper alloys in the semi-solid state.
- Identification of shear-thinning copper alloys’ behaviour in the semi-solid state.
- Identification of thixotropic phenomena in copper alloys in the semi-solid state.
- Identification of Bingham behaviour of copper alloys in the semi-solid state.

**Abstract:**

The analysis of rheological properties provides information that allows the potential for the practical application of metal alloys to be determined and to effectively design their shaping with the use of advanced engineering modelling. The paper presents an analysis of the basic rheological properties of selected copper alloys, CuSn10 and CuAl10Fe3Mn2, in the liquid and semi-solid states. The analysis was based on viscosity measurements and rheological modelling. The measurements were carried out using a high-temperature rheometer with the Searle method. The research described in this article was undertaken to provide data for assessing the potential of using thixoforming technologies to shape copper matrix alloys; research data in this field are insufficient and information in the literature is also limited. The analysis carried out includes the complex characterisation of viscosity in terms of the influence of temperature, strain rate and time. The results were enriched with rheological models that can be used in simulation studies.

## 1. Introduction

Copper alloys belong to one of the basic metal groups characterised by a wide range of applications in engineering, electrical, construction and maritime industries; the wide range of applications is due to the many advantages offered by these alloys. Four basic features deserve attention here: good mechanical properties, high corrosion resistance, high electrical conductivity and thermal conductivity.

The rheological studies reported in the literature concern primarily steels, other high-melting-point alloys [1,2,3,4,5,6], and light metal alloys [7,8,9,10,11,12,13,14,15,16,17,18,19,20,21,22,23,24,25,26,27,28,29,30] processed in the semi-solid state. This is further supported by rheological characterisation studies in the form of viscosity measurements, globular microstructure development analysis, and thixoforming process design. However, despite the growing interest in semi-solid state processing technologies, similar studies on copper alloys remain limited. For example, S.J. Harboe [24] described the behaviour of a semi-solid Al–Cu alloy subjected to isothermal shear. Studies have shown that the change in apparent viscosity with changing shear rate is related to the disintegration or agglomeration of the solid-phase particles. A decrease in shear rate leads to partial agglomeration of the solid particle network. This close relationship between the rheological response and changes in microstructure supports the interpretation of shear thinning of the semi-solid alloy, and the time-dependent viscosity confirms the phenomenon of thixotropy. Therefore, this paper focuses on the rheological behaviour of selected copper alloys in the semi-solid state.

Copper alloys, like most other metals, can be shaped by casting and metal forming technologies but, as a rule, casting processes do not guarantee products with comparable strength to those produced by metal forming processes. This relates to the total accumulated deformation of the material in the solid state, which guarantees the absence of porosity and strengthened grains in the microstructure. Qualification of copper alloys for shaping processes is based on the criterion of material plasticity, with less ductile alloys being cast, while highly ductile alloys are shaped using metal forming techniques. However, there is a third, but rarely applied, method of forming metal alloys that involves processing in the semi-solid state [31,32]. This method, under the generic term of thixoforming, is used to shape alloys with a wide temperature range between the solidus and liquidus points. From a technological point of view, thixoforming is typically carried out using modified high-pressure die-casting machines or forging presses, with the former variant called thixocasting and the latter called thixoforging.

Thixoforming processes are mainly intended for alloys characterised by low plasticity and, at the same time, possessing appropriate solidification parameters to technologically maintain the semi-solid state during this process. As a result, part of this work is concerned with the identification of copper alloys that meet the above assumptions.

**Figure 1:**
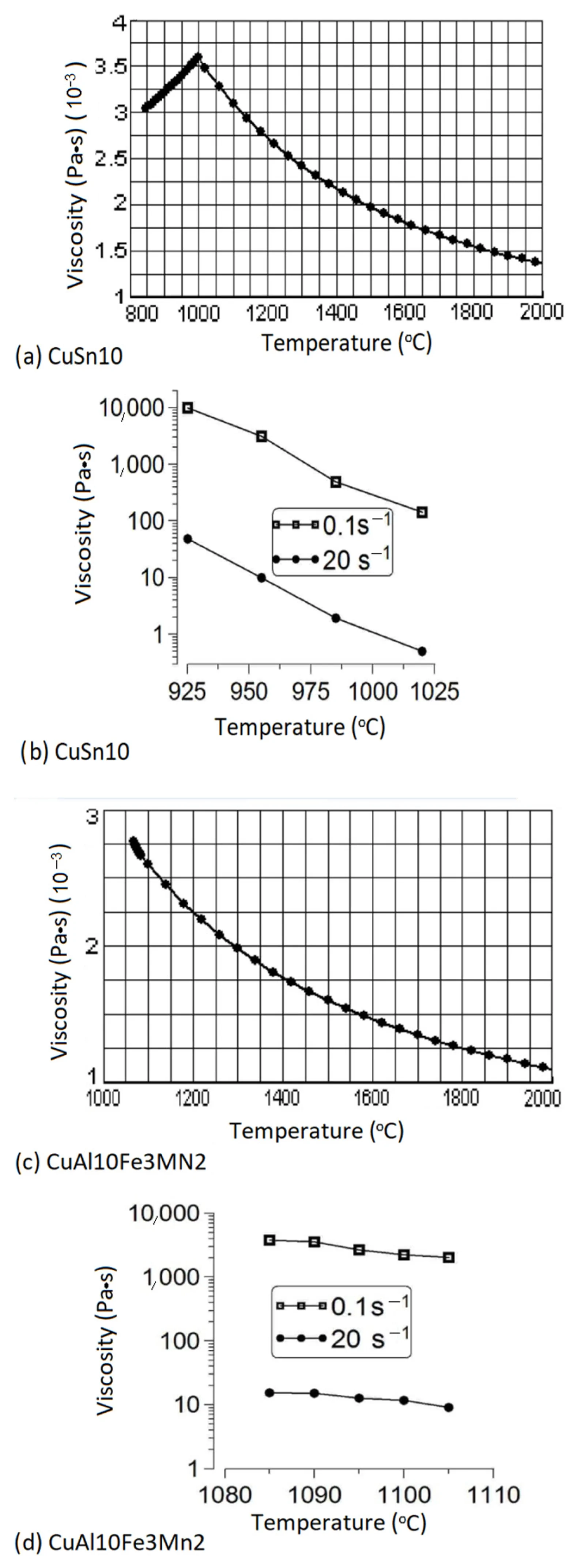
Comparison of viscosity as a function of temperature obtained by calculations using ProCAST software (**a**,**c**) and from experimental measurements (**b**,**d**) for CuSn10 and CuAl10Fe3Mn2 alloys.

The study of the rheological properties of a selected copper alloy in the semi-solid state has two basic objectives: first, the selection of thixoforming process parameters and, second, expanding our rheological knowledge of copper alloys in the semi-solid state. At present, the shaping of copper alloys is carried out very rarely in the semi-solid state on an industrial scale, and the research conducted in this work aims to prove the potential of possible implementation of such processes. The rheological properties of low-melting-point metal alloys and steels in the semi-solid state are described in detail in the current literature [33,34,35,36,37]. However, it should be noted that, currently, there is not much information on the rheology of copper alloys in the semi-solid state. Mathematical models, especially those supported using digital machines, is a relatively cheap and effective tool for designing and optimising industrial processes, but numerical packages require application of accurate information describing the rheology of the process at hand. Some models have implemented thermodynamic databases, but these do not provide a precise enough rheological description. One of the reasons for undertaking this research is explained in Figure 1.

Figure 1 shows a comparison of viscosity as a function of temperature obtained from experimental measurements and from calculations using the ProCAST software (ver. 2016.0) for CuSn10 and CuAl10Fe3Mn2 alloys. As part of the research, measurements were performed of the dynamic viscosity of CuSn10 and CuAl10Fe3Mn2 copper alloys. The description of rheological properties most often used to model thixoforming processes is in the form of a coefficient of dynamic viscosity. This coefficient is a function of several parameters, the most important of which are temperature, shear rate and time. The influence of temperature is particularly strong, especially in the solidification temperature range where phase changes cause an increase in the fraction of the solid phase during cooling, resulting in a significant increase in viscosity. The second parameter that significantly affects the viscosity is shear rate, indicating that semi-solid copper alloys are non-Newtonian liquids. The work also identified the non-Newtonian properties of copper alloys resulting from the phenomenon of shear thinning. This phenomenon causes a decrease in the viscosity value with an increase in the shear rate. The third parameter influencing the viscosity is time of deformation and, in the semi-solid state, the phenomenon of thixotropy occurs that causes the viscosity to decrease during the time of deformation.

In order to carry out the various measurements, a number of different devices were used in this work, such as a high-temperature rheometer with a furnace that allows measurements of the rheological properties of metal alloys in the range from 400 to 1500 °C and a Zwick HB100 hydraulic machine, used to determine the plasticity of selected copper alloys, designed to perform static and dynamic tests in the range of forces +/− 100 kN. Due to its modular configuration, the Zwick can be adapted to any sample geometry, allowing a number of static tensile tests to be performed for the alloys analysed. These studies provided the information that allowed the selection of a low-ductility aluminium bronze that could be potentially formed in the semi-solid state. In order to determine the fraction of the solid phase as a function of solidification temperature, thermodynamic databases of the ProCAST package were used in the work. This relationship is necessary to determine one of the internal parameters of rheological models and the appropriate viscosity measurement temperatures. Finally, the Grapher application, which has built-in approximation tools, was used to identify rheological parameters and prepare the various graphs included in this article.

## 2. Materials for Research

Copper alloys are typically divided into brasses and bronzes. Brasses are alloys of copper and zinc. These alloys contain about 46–97% of copper. Special brasses contain, in addition to copper and zinc, manganese, lead, iron, aluminium, silicon, nickel and tin. The mechanical properties of brasses depend on the zinc content, the strength and ductility increasing with increasing zinc content. Brasses are divided into foundry and plastic forming alloys. Foundry brasses are multi-component alloys that contain silicon up to 4.5%, manganese up to 4%, lead up to 4%, aluminium up to 3% and iron up to 1.5%. Lead and silicon improve castability. Zinc, aluminium, manganese and iron increase strength properties. Wrought brasses have a lower content of alloying elements than foundry brasses, because the increase in content of some alloying elements causes a decrease in plastic properties of the alloys. Bronzes are also divided into foundry and wrought copper alloys, classified by the name of the main alloying element; i.e., aluminium, beryllium, tin, silicon or manganese bronzes.

In general, brasses are more ductile and have a narrower range of solidification temperatures than bronzes; therefore, thixoforming processes are naturally more suitable for shaping bronzes. This paper investigated the mechanical properties of a series of bronzes with particular emphasis on their plasticity. These alloys were purchased from commercial suppliers, and their mechanical properties were investigated, using static tensile tests, carried out on a Zwick HB100 testing machine (ZwicRoell, Ülm, Germany). Table 1 shows the test results for different groups of bronzes comparing their mechanical properties.

The processing of metal alloys in the semi-solid state requires a wide range of solidification temperatures. This requirement results from the inability to maintain perfectly isothermal conditions in an industrial setup, especially at temperatures above 1000 °C. To assess the feasibility of subjecting metal alloys to shaping in the semi-solid state, criteria are used that would support the practicability of using the process, relating to the stability of the semi-solid state. Among these criteria are the temperature difference between the solidus and liquidus points and the temperature range size for the liquid phase fraction between 20% and 60% or, for example, the derivative of the liquid phase fraction by the temperature for a 40% liquid phase fraction. From the technological point of view for copper alloys, it could be assumed that the temperature difference between the solidus and liquidus points should be at least 60 °C. The size of the temperature ranges for the fraction of the liquid phase in the range from 20% to 60% should be at least 30 °C, and the derivative of the fraction of the liquid phase by temperature should be less than 0.02.

**Figure 2:**
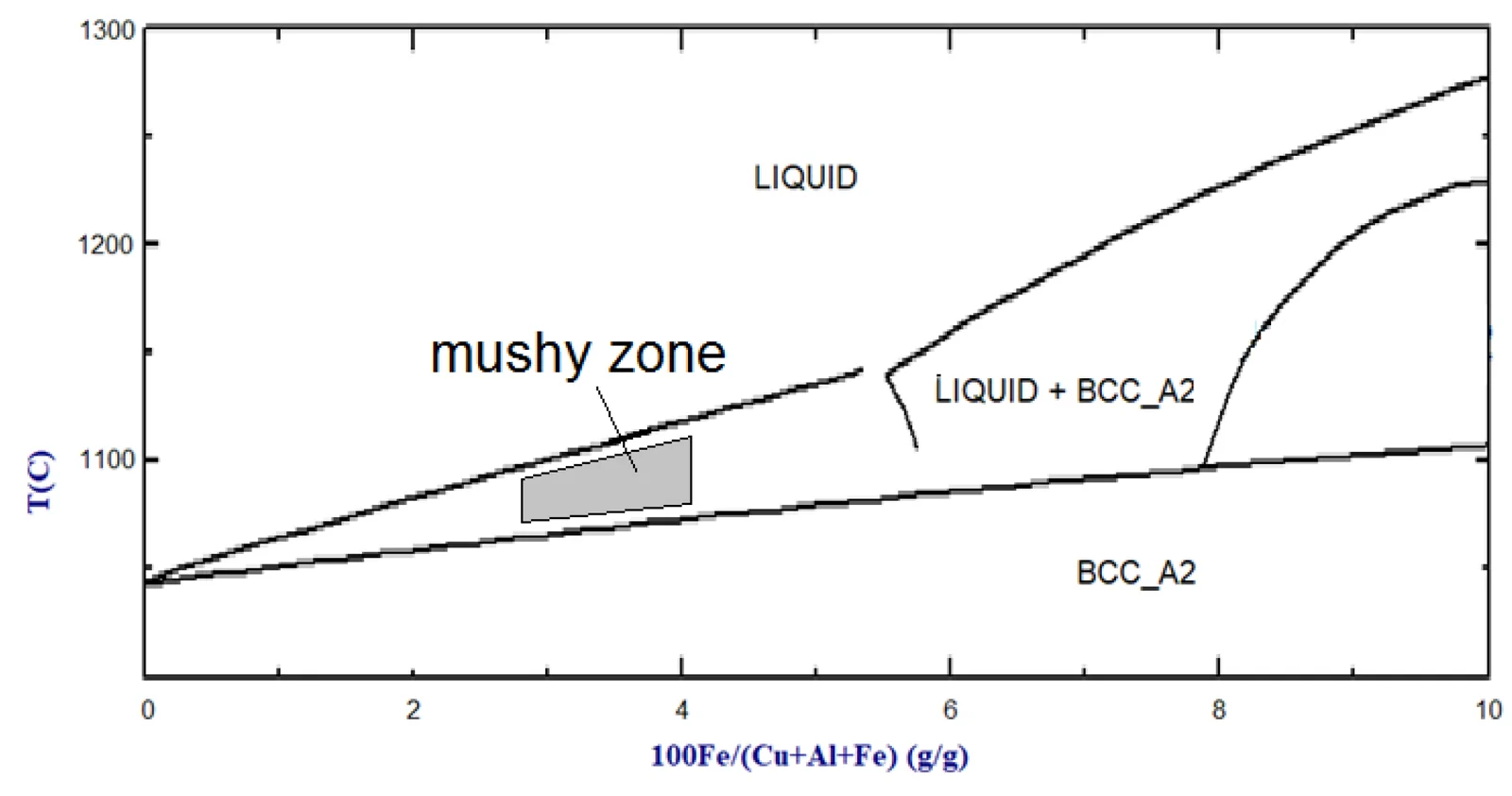
Binary phase diagram of copper–ferrous alloy including 10% aluminium (FactSage GTT-Technologies, Herzogenrath, Germany).

In this work, several phase systems of copper with other elements were analysed and simulations of the fraction of the liquid phase as a function of temperature were performed for a few commercial alloys. The analysis shows that a wide range of solidification temperatures occurs in alloys of copper with tin, aluminium, silicon and beryllium. The analysis of the properties of alloys with beryllium was rejected due to the high toxicity of these alloys in the liquid state. These alloys contain between 1.6% and 2.1% beryllium. Among the aluminium bronzes, the alloy BA1032 (CuAl10Fe3Mn2) was a potential candidate. This alloy has a poor range of only about 60 °C from 1050 to 1110 °C. Copper–silicon alloys have interesting properties; for example, the CuSi3 alloy has a solidification range of about 70 °C from 965 to 1035 °C. Another alloy that was analysed was the CuSn10 tin alloy that was selected for testing because it is a casting alloy with relatively low plasticity. This alloy has a very wide range of solidification temperatures of up to about 160 °C from 837 to 994 °C. Some of the aluminium and silicon bronzes were rejected because they have small solidification temperature ranges and, at the same time, they are alloys intended for plastic processing. Figure 2 shows the binary phase diagram of a copper–ferrous alloy including 10% aluminium, corresponding to CuAl10Fe3Mn2, with an appropriate range of temperatures and chemical composition for semi-solid processing.

The analysis of solidification parameters shown in Table 1 allows identification of copper alloys appropriate for the thixoforming process. It is obvious that most tin bronzes and the CuSi3 bronze belong to this group. Moreover, some aluminium bronzes, such as the CuAl10Fe3Mn2 bronze, could also be considered as possible candidates. In this paper the rheological properties of CuSn10 and CuAl10Fe3Mn2 alloys were analysed as they have appropriate properties and have been selected by other authors as potential candidates for the thixoforming processes [38].

According to previously established assumptions, the CuSn10 and CuAl10Fe3Mn2 alloys meet the technological requirements, i.e., the solidification range exceeds 60 °C, the temperature range corresponding to the liquid phase fraction range between 20 and 60% exceeds 30 °C, and the temperature derivative of the liquid phase fraction is less than 0.02.

## 3. Research Methodology

The viscosity of the analysed copper alloys was measured on a high-temperature rheometer, FRS 1600 (Anton Paar GmbH, Gräz, Austria), equipped with a furnace, enabling the samples to be heated to temperatures ranging between 400 and1500 °C. Measurements can be carried out using various control parameters; most often, the shear rate, rotational speed, shear stress or torque are used for this purpose. The research was carried out using the Searle method. In industrial laboratories, most rheometers perform measurements based on the method developed by G.F.C. Searle in 1912 [39,40]. One disadvantage of this method is the risk of turbulent flow of low-viscosity liquids during the measurement at high rotational speeds, when the so-called “Taylor vortices” are created, caused by centrifugal forces and the phenomenon of inertia in liquids.

**Figure 3:**
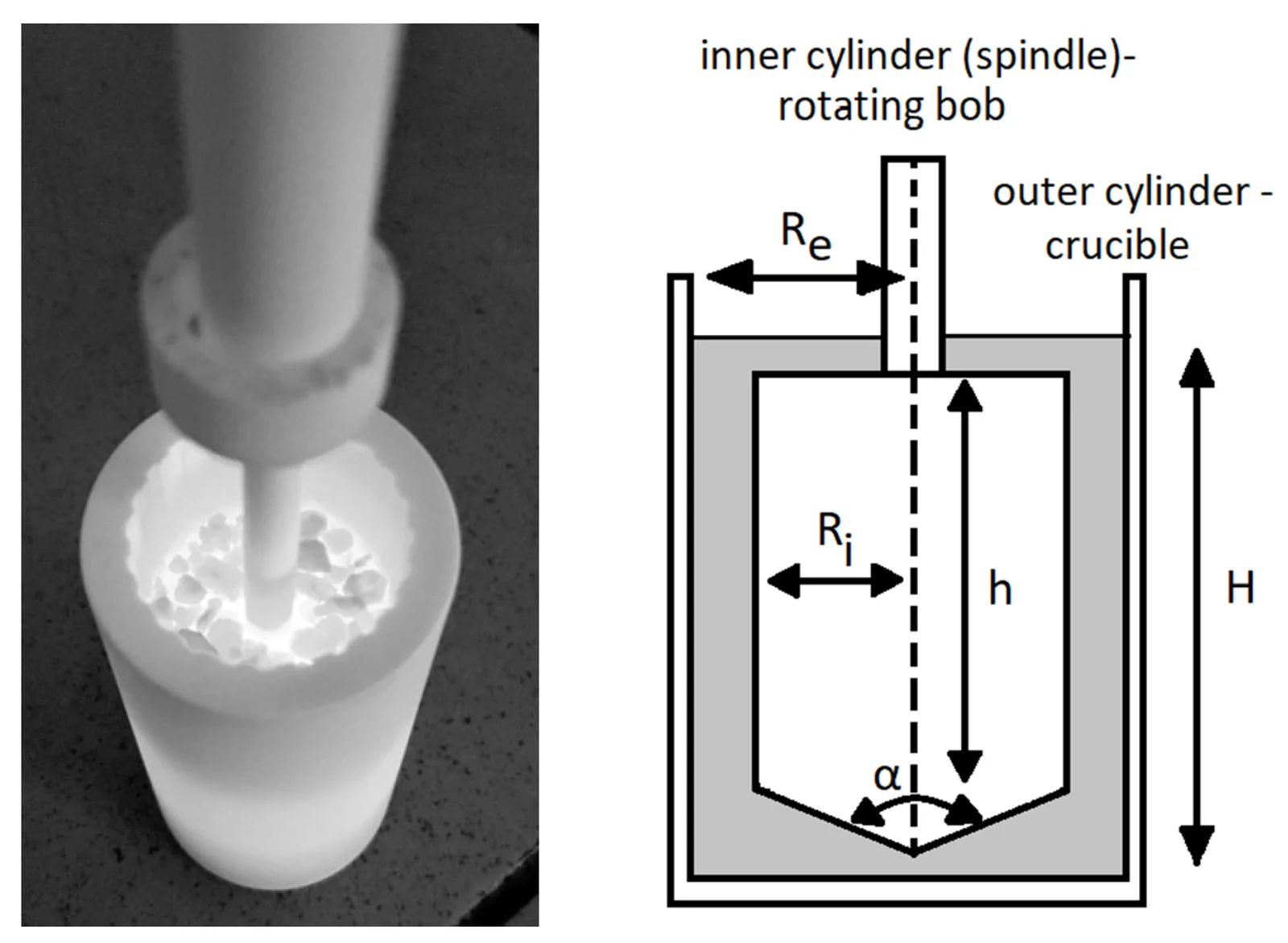
Photograph and schematic arrangement of the measurement tools with dimensions—two cylinders; spindle—movable inner cylinder; and crucible—stationary outer cylinder.

The Searle method uses two cylinders, the first is the so-called spindle—a movable inner cylinder—and the second is the crucible—a stationary outer cylinder. The measurement arrangements are shown schematically in Figure 3. The cylinders are concentric, which means that they have a common axis of symmetry, which is also the axis of rotation of the spindle.

In this work the yield stress of investigated alloys was also determined. The estimation procedure was like the method described by Harboe and Modigell [20,21,41]. A shear stress ramp method was used to determine the yield stress. Measurements were conducted using a coaxial cylinder geometry. A protective gas containing 99.9% argon was also used during the experiment to prevent sample oxidation. Prior to the actual measurement, the samples were heated until fully molten. The material was then homogenised by shearing in the liquid state for 15 min. Following homogenisation, the samples were cooled at a rate of 5 °C/min under continuous shear to temperatures corresponding to the target solid fraction. Upon reaching the measurement temperature, the material was subjected to further shearing for half an hour to break down dendrite arms and achieve a globular structure. A constant shear rate of 20 s^−1^ was applied during material preparation. After sample preparation, a pre-shear stage was performed, in which the material was subjected to a constant shear rate for a specified duration. Depending on the experimental variant, the shear rate during this stage was either 10 or 20 s^−1^. The pre-shear stage was followed by a rest period during which the material was not subjected to shear. The rest duration was varied to determine the dynamic yield stress value; in specific experiments, its duration was dictated by the rate of shear stress increase employed. During the ramp, the shear stress was increased linearly at a constant rate, while the material’s response—in the form of the shear rate—was simultaneously recorded. The yield stress was determined based on the sudden increase in shear rate observed once the stress exceeded the critical threshold. The shear stress corresponding to the sudden increase in shear rate was taken as the yield stress value, *τ*_0_.

The geometrical parameters of the system were as follows: *R_i_* = 10 mm, *R_e_* = 13 mm, *H* = 30 mm, and *h* = 20 mm. In the method of measuring viscosity using a concentric cylinder arrangement, the flow parameters are defined as a function of the radii of these cylinders. The representative shear stress is defined as follows:

(1) $$\tau =\tau_{\mathrm{rep}}=\;\frac{1+\delta_{\mathrm{cc}}^{2}}{2\delta_{\mathrm{cc}}^{2}}\frac{M}{2\mathrm{\pi L}R_{i}^{2}c_{L}}=\;C_{\mathrm{ss}}M$$

(2) $$\delta_{\mathrm{cc}}=\frac{R_{e}}{R_{i}}$$

where *M* [Nm] is torque on the surface of the inner cylinder, *h* [m] is the length of the inner cylinder, *C_L_* [-] is the torque correction factor (at the tip of the cone of the inner cylinder), *C_ss_* [Pa/Nm;m^−3^] is the constant of the concentric cylinder measurement system, *Css* depends only on the geometrical parameters in the concentric cylinder method, i.e., *R_i_*, *δ_cc_* [-] is the ratio of the outer cylinder radius to the inner cylinder radius, *R_e_* [m] is value of the radius of the outer cylinder and *R_i_* [m] is the value of the radius of the inner cylinder.

The representative shear rate is defined by formula (3).

(3) $$\dot{\gamma }={\dot{\gamma }}_{\mathrm{rep}}=\frac{\left(1+\delta_{\mathrm{cc}}^{2}\right)}{\delta_{\mathrm{cc}}^{2}-1}\omega =C_{\mathrm{sr}}\cdot n$$

where *ω* [rad/s] is angular velocity, *n* [min^−1^] is number of revolutions per minute, and *C_sr_* [min/s] is a constant of the measuring system. *C_sr_* depends on the ratio of the radius of the inner cylinder *R_i_* to the radius of the outer cylinder *R_e_*.

For the geometry of the system shown in Figure 3, the values of *C_ss_* and *C_sr_* calculated from Formulas (1) and (3) are, respectively: 54,042.04 Pa/Nm (the assumed value of the coefficient *C_L_* = 0.85) and 24.49 s/s.

## 4. Experimental Work

As part of the experimental work, measurements of viscosity as a function of temperature and shear rate were performed. The measurements were made using the Anton Paar model FRS 1600 rheometer. First, the dependence of viscosity as a function of temperature was determined and the resulting measurements are shown in Figure 1b,d. The measurement program implemented in the Rheoplus software (ver. 3.40) changed the temperature values during the shearing of the sample. Measurements were made for different temperatures corresponding to the semi-solid state.

**Figure 4:**
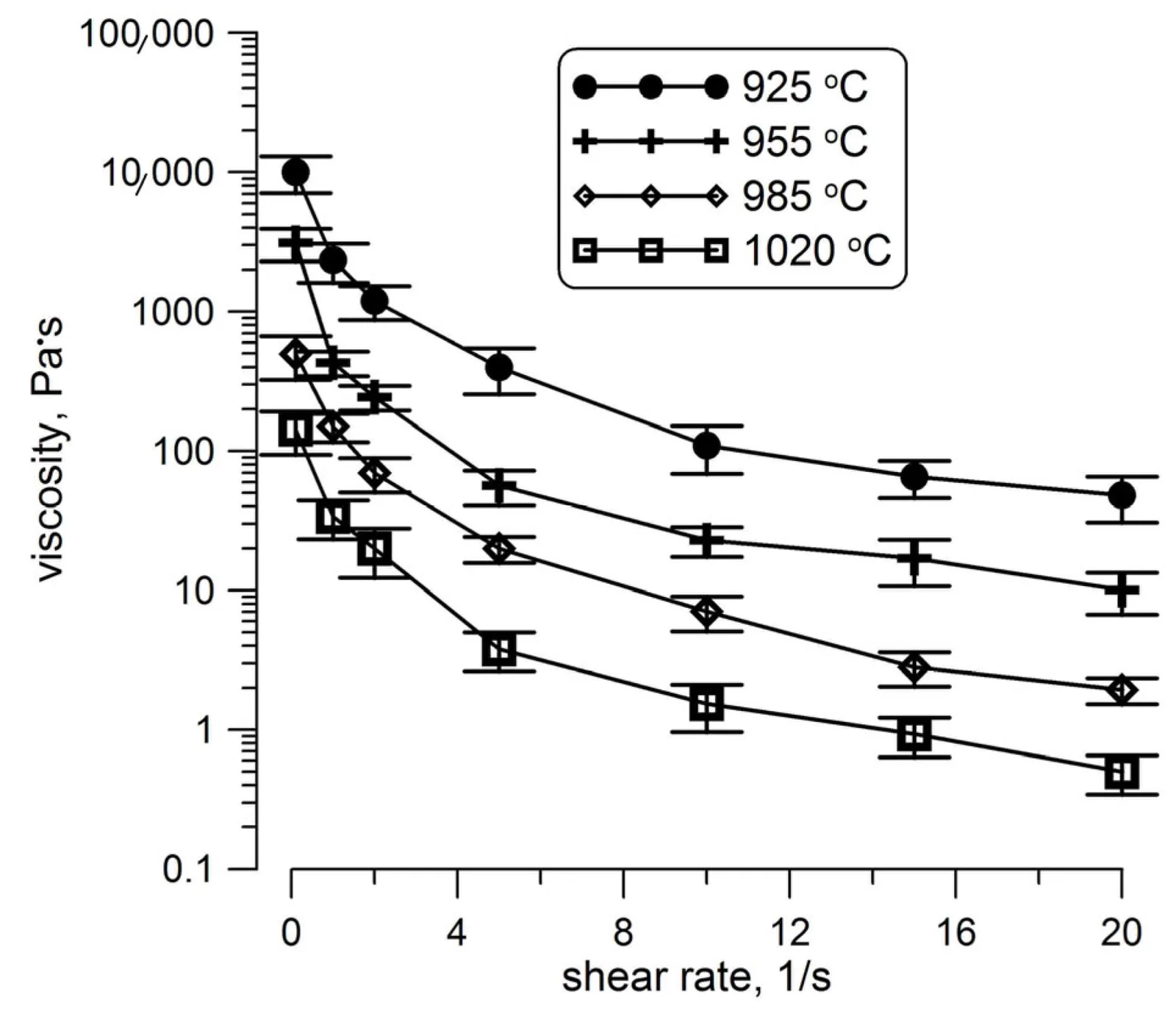
Dependence of viscosity as a function of shear rate for different temperature values for CuSn10.

**Figure 5:**
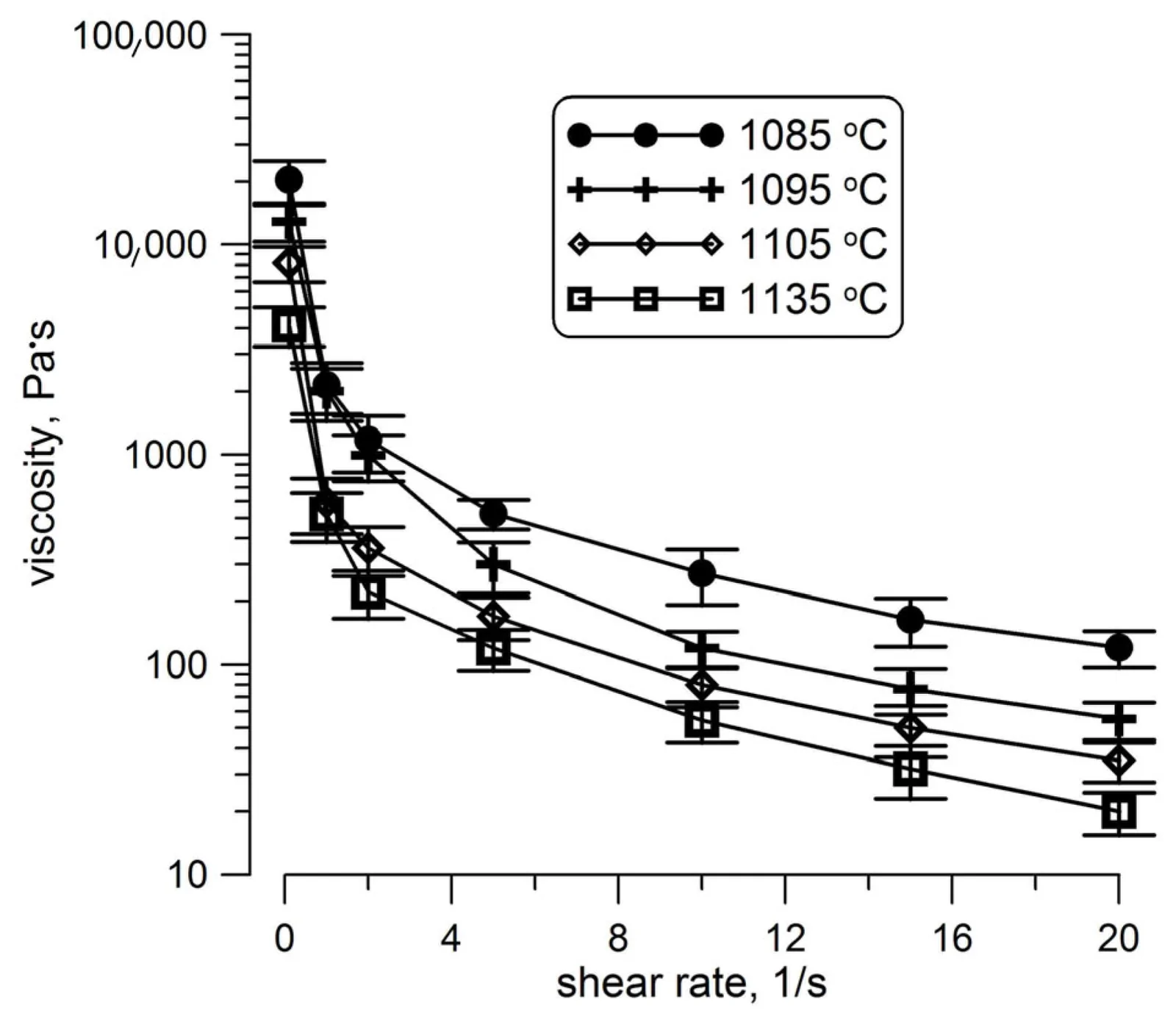
Dependence of viscosity as a function of shear rate for different temperature values for CuAl10Fe3Mn2.

In the next step, the influence of shear rate on viscosity was determined. The measurements were programmed in such a way that the value of the shear rate changed for a constant sample temperature. In this way, viscosity values were determined for shear rates of 0.1, 1, 2, 5, 10, 15 and 20 s^−1^. The results of the measurements are shown in Figure 4 and Figure 5. Finally, the curves of viscosity as a function of shear rate were determined for four different temperatures. Error bars were also added to assess measurement errors. The errors were determined based on several measurements performed using the same instrument settings.

**Figure 6:**
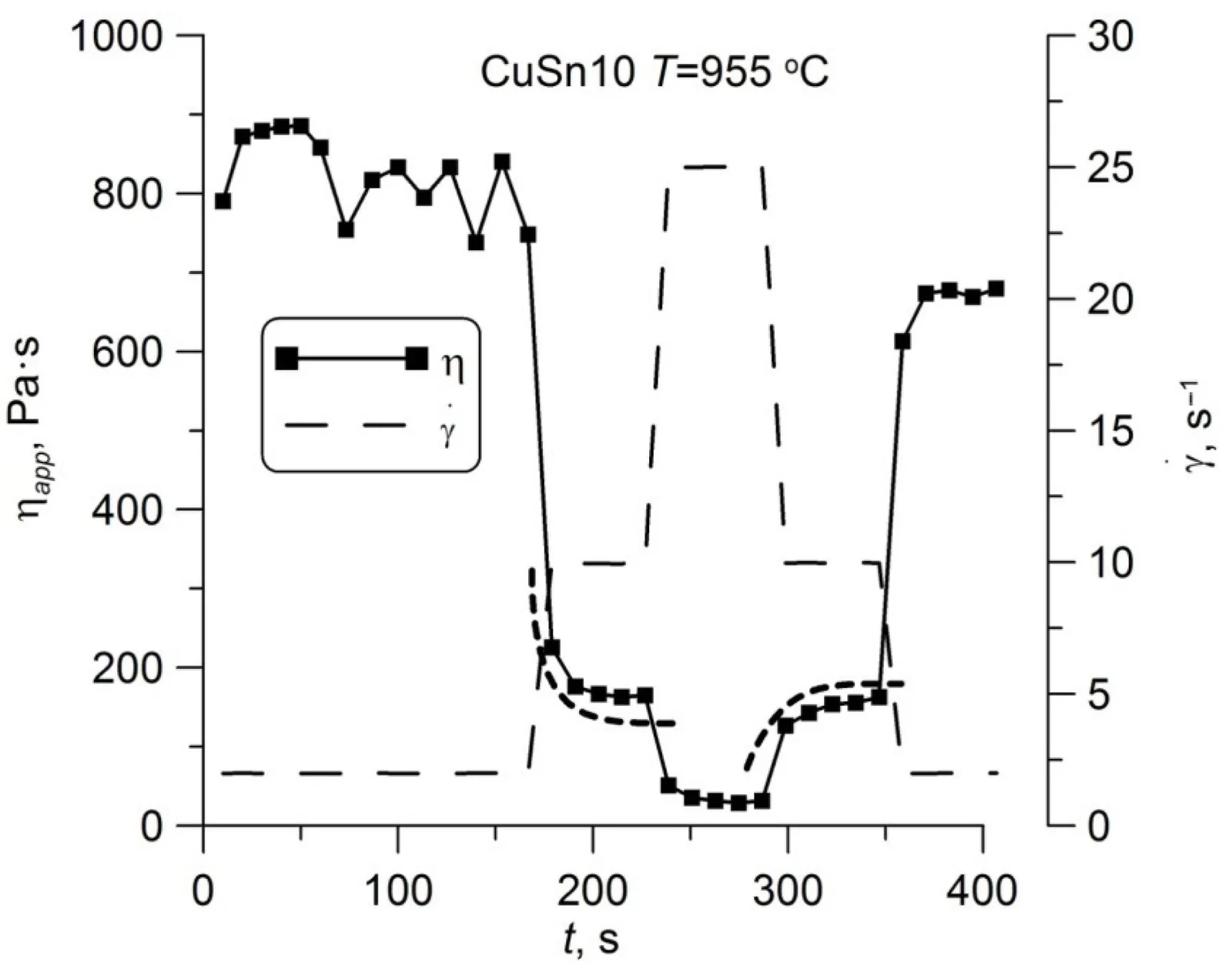
Viscosity versus time for constant values of temperature and shear rate for CuSn10 alloy.

**Figure 7:**
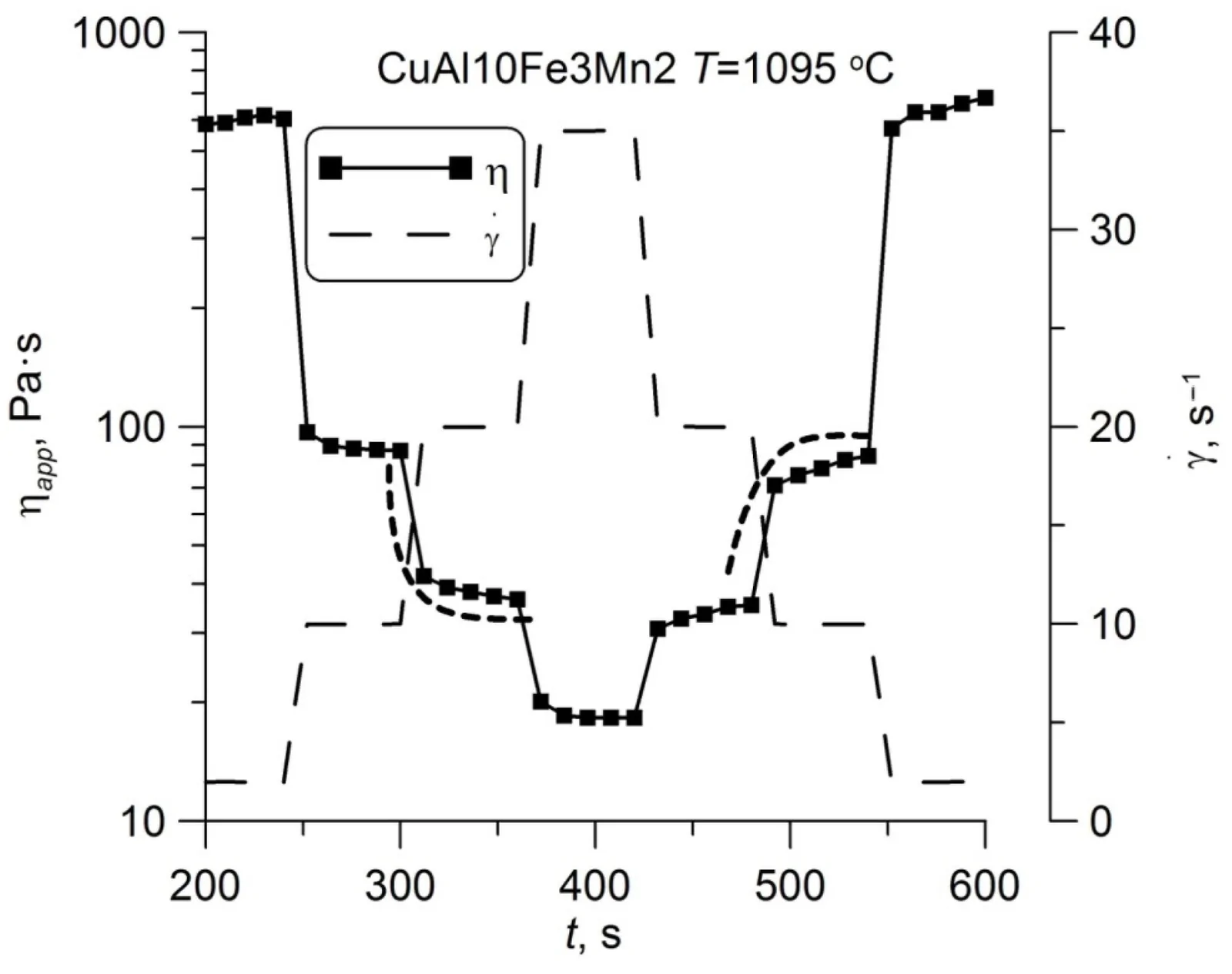
Viscosity versus time for constant values of temperature and shear rate for CuAl10Fe3Mn2 alloy.

Next, measurements of viscosity versus time for constant values of temperature and shear rate are shown in Figure 6 and Figure 7 for CuSn10 and CuAl10Fe3Mn2 alloys, respectively.

**Figure 8:**
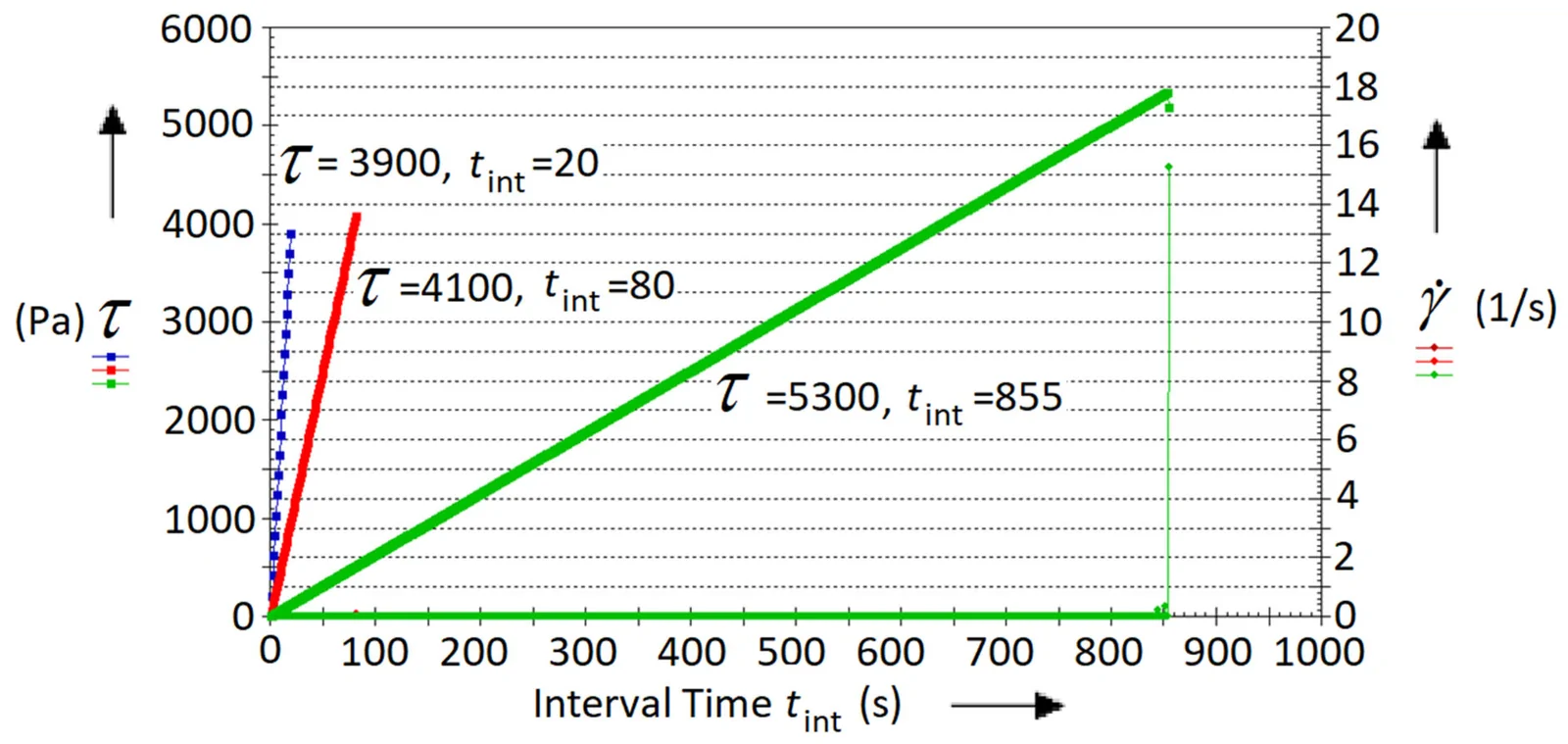
Yield stress values versus interval time (rest time) for CuAl10Fe3Mn2 alloy for 1080 °C.

**Figure 9:**
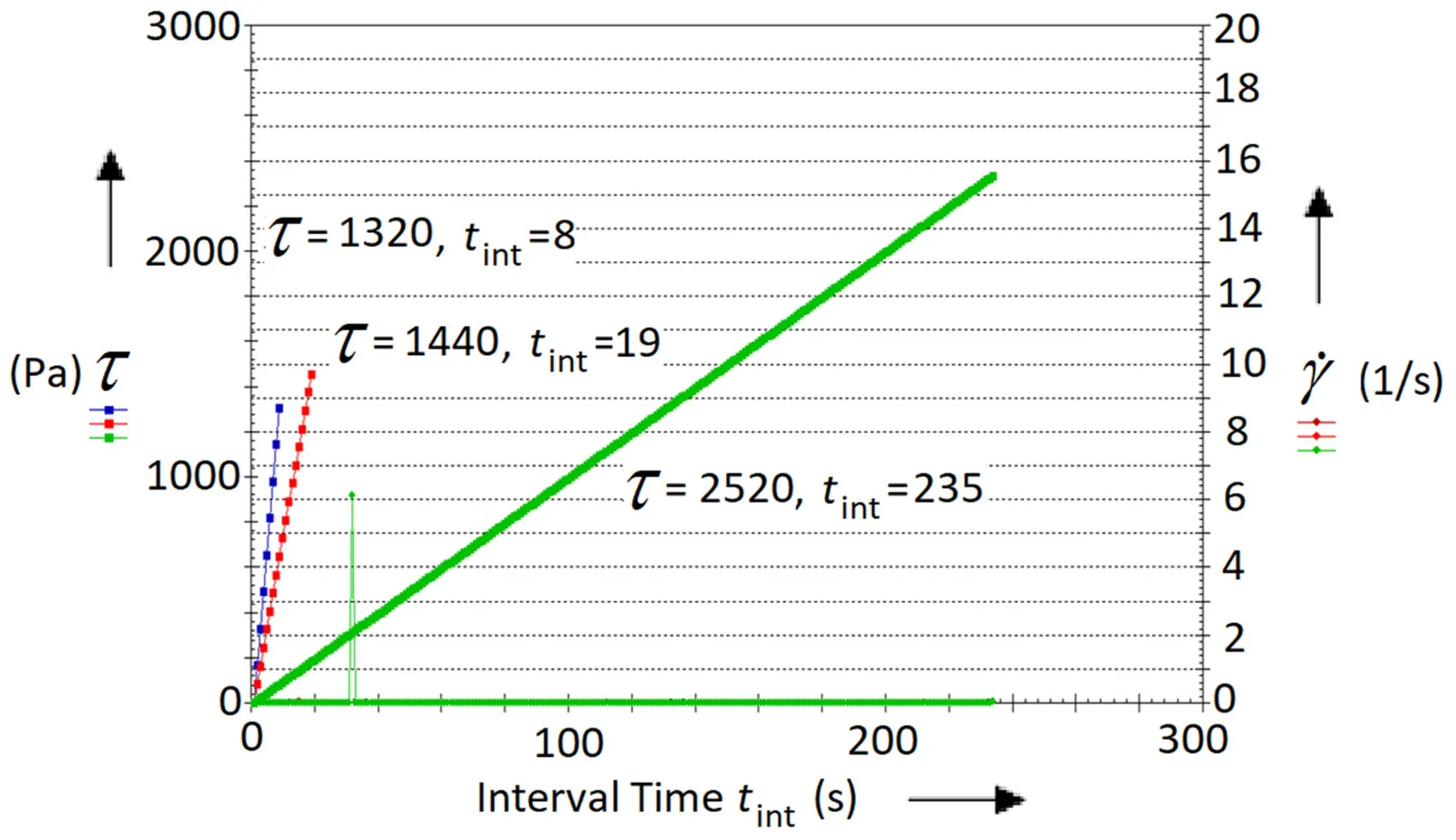
Yield stress values versus interval time (rest time) for CuAl10Fe3Mn2 alloy for 1090 °C.

Finally, the yield stress for the investigated alloys was experimentally identified. The yield stress was measured after varying rest times between stages of deformation. Figure 8 and Figure 9 show this relationship for CuAl10Fe3Mn2 alloy at two different temperatures.

## 5. Rheology Modelling

The rheological measurements were used in the later stage of the work to develop rheological models that can be applied to the design and modelling of the thixoforming processes for theCuSn10 and CuAl10Fe3Mn2 alloys. Generally, Computational Fluid Dynamics (CFD) class modelling programs use different classes of rheological models that, in the simplest terms, treat liquids as Newtonian fluids whose viscosity does not depend on temperature and shear rate but more advanced thermo-mechanical models have viscosity as dependent on temperature. However, the most popular option is models that, apart from temperature, also consider the effect of shear rate. As a result, models with different complexity of describing the viscosity of the analysed alloys were developed in this work.

First, for the measured viscosity curves, a temperature-dependent model was used, represented by Equation (4) [42].

*η*
(*T*) = *A*exp(*B*/*T*) (4)

where *T* is temperature and *A*, *B* are the rheological parameters.

The values of rheological parameters for all shear rate values for which the dependence of viscosity on temperature was measured are shown in Table 2.

The correctness of approximation was assessed using the coefficient of determination, *R*^2^, which is a measure used in statistics and regression analysis that indicates how well a regression model explains the variation in the dependent variable (in this case, viscosity *η*) based on the independent variables ($\dot{\gamma }$). The *R*^2^ value ranges from 0 to 1. A value of one indicates that the model perfectly predicts the viscosity, whereas a value of zero indicates that the model does not explain the variation at all. Thus, the higher the coefficient of determination, the better the model fits the measurement data. The fit of model (4) was evaluated based on the coefficient of determination (*R*^2^); values for the individual shear rates are presented in Table 2.

**Figure 10:**
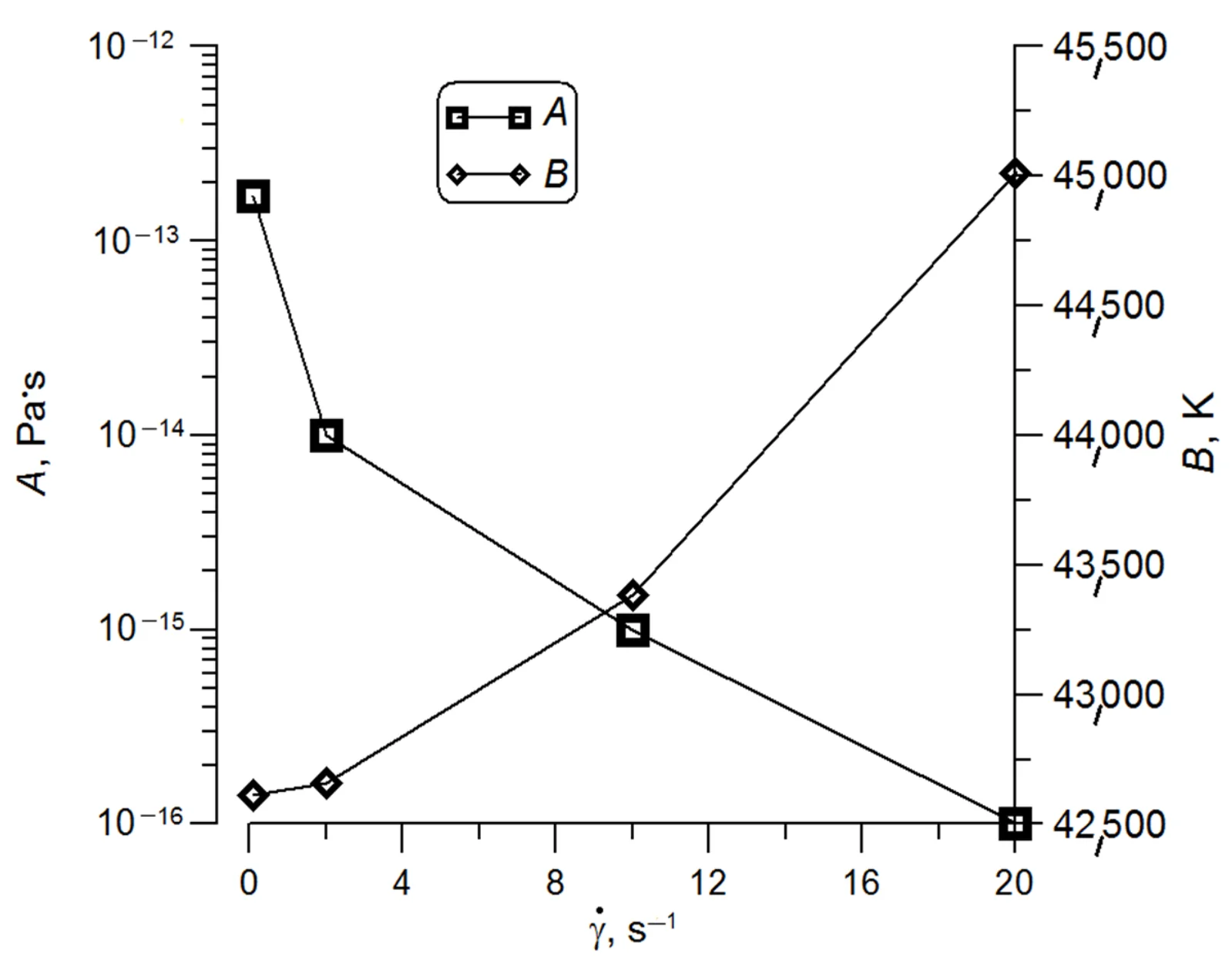
Graph of parameter values of rheological model (4) versus shear rate for CuAl10Fe3Mn2.

**Figure 11:**
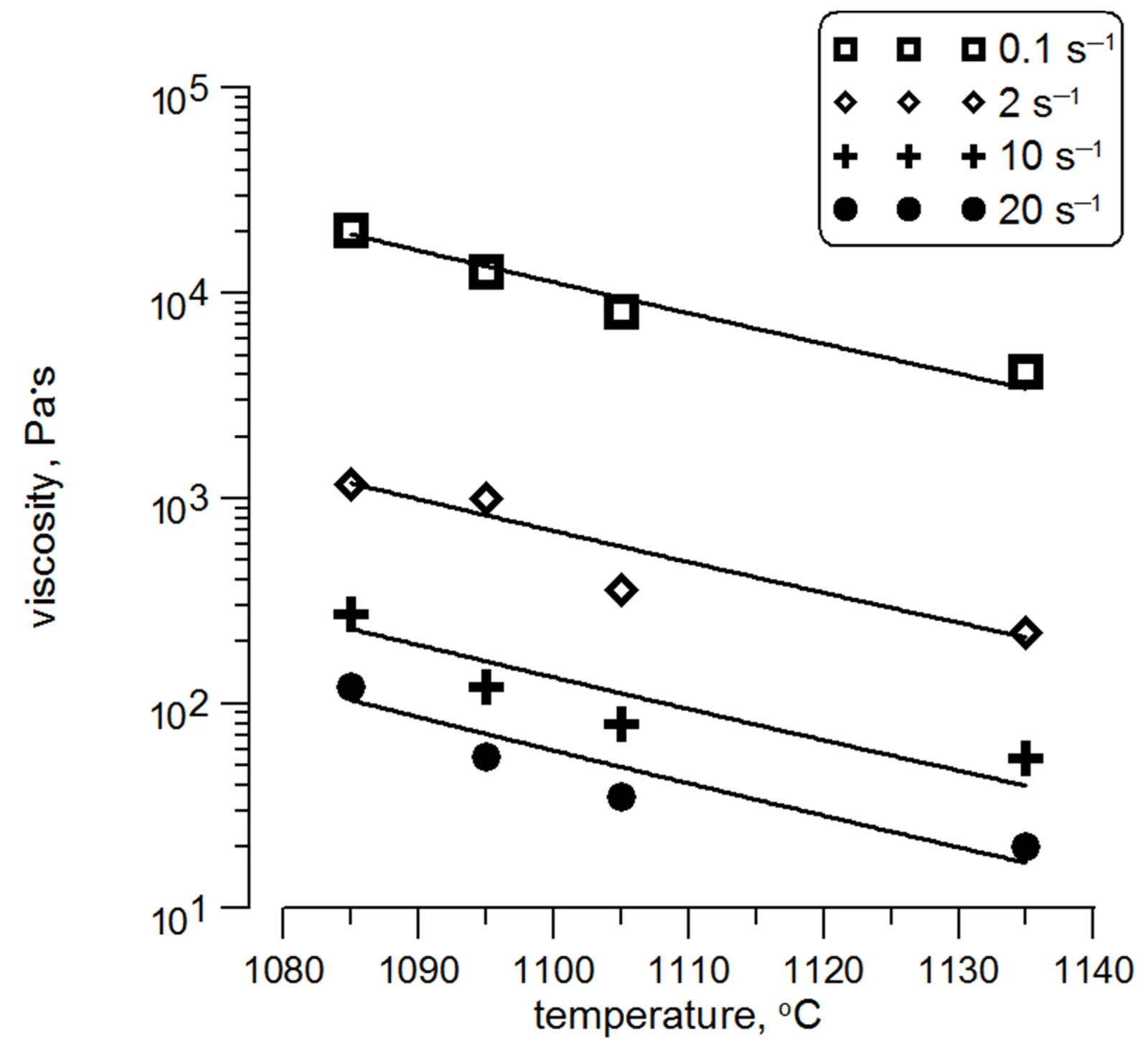
Graph of viscosity versus temperature calculated using model (4) with points showing measured values of viscosity for CuAl10Fe3Mn2.

Figure 10 shows the graphs of rheological parameters of model (4) as a function of shear rate. Figure 11 shows the graph of viscosity as a function of temperature, calculated using model (4) with marked measurement points. This plot shows the accuracy of the approximation using this model.

A rheological equation often used in simulations is the Carreau–Yasuda model (5) [43]. This model describes viscosity as a function of shear rate, thus allowing modelling of metal alloys as non-Newtonian fluids. If, in addition, the simulation software allows for using many sets of rheological parameters for different temperatures, then a dependence is also obtained on the temperature.

(5) $$\frac{\eta -\eta_{\infty }}{\eta_{0}-\eta_{\infty }}={\left[1+{\left(\lambda \dot{\gamma }\right)}^{a}\right]}^{\frac{n-1}{a}}$$

where $\dot{\gamma }$ is the shear rate and *η*_0_, η_∞_, λ, *a*, *n* are the rheological parameters.

Table 3 presents the values of rheological parameters for all temperature values for which the dependence of viscosity on shear rate was measured. The fit of model (5) was evaluated based on the coefficient of determination (*R*^2^); values for the individual temperatures are presented in Table 3.

**Figure 12:**
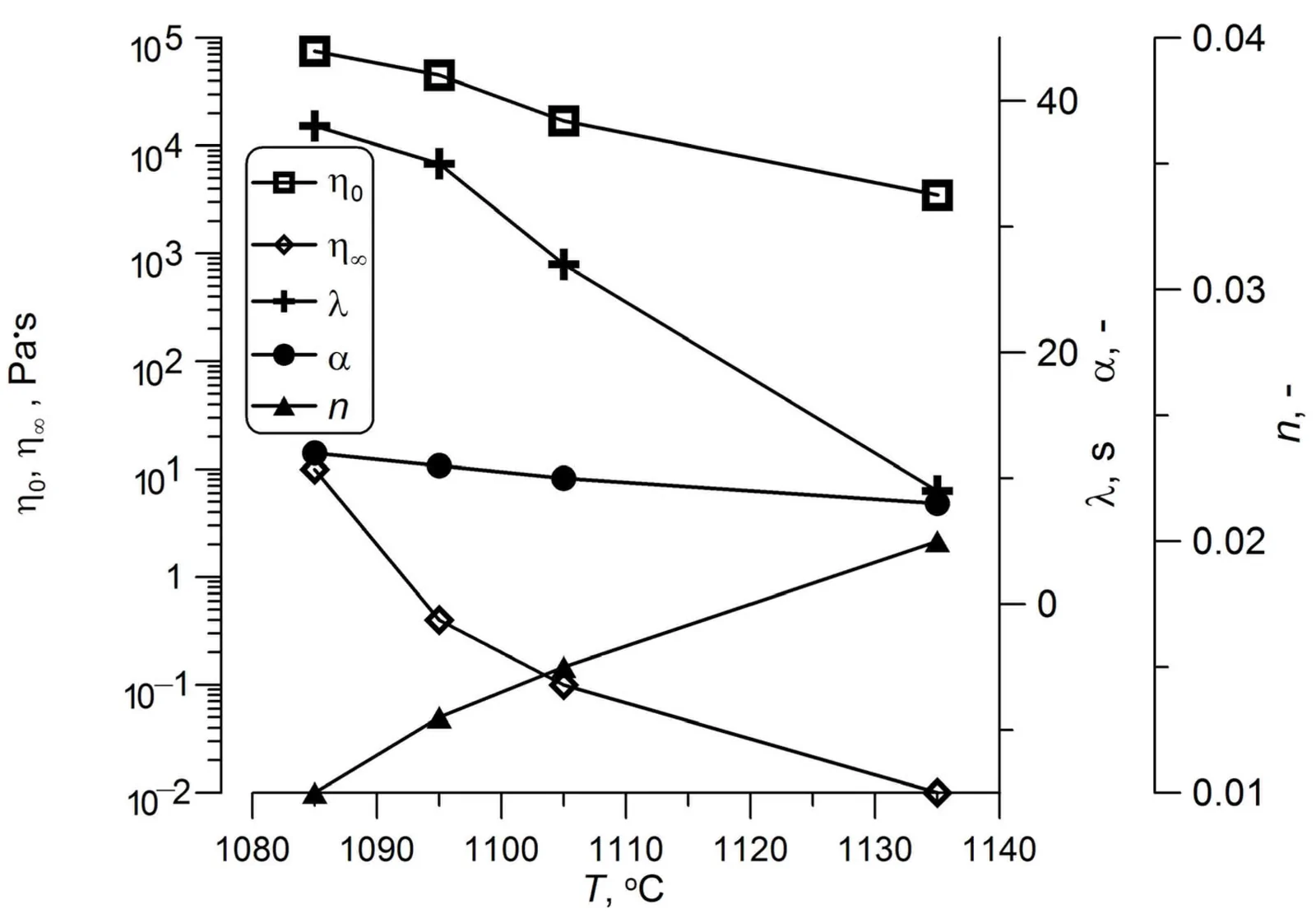
Graph of parameter values of rheological model (5) versus shear rate for CuAl10Fe3Mn2.

**Figure 13:**
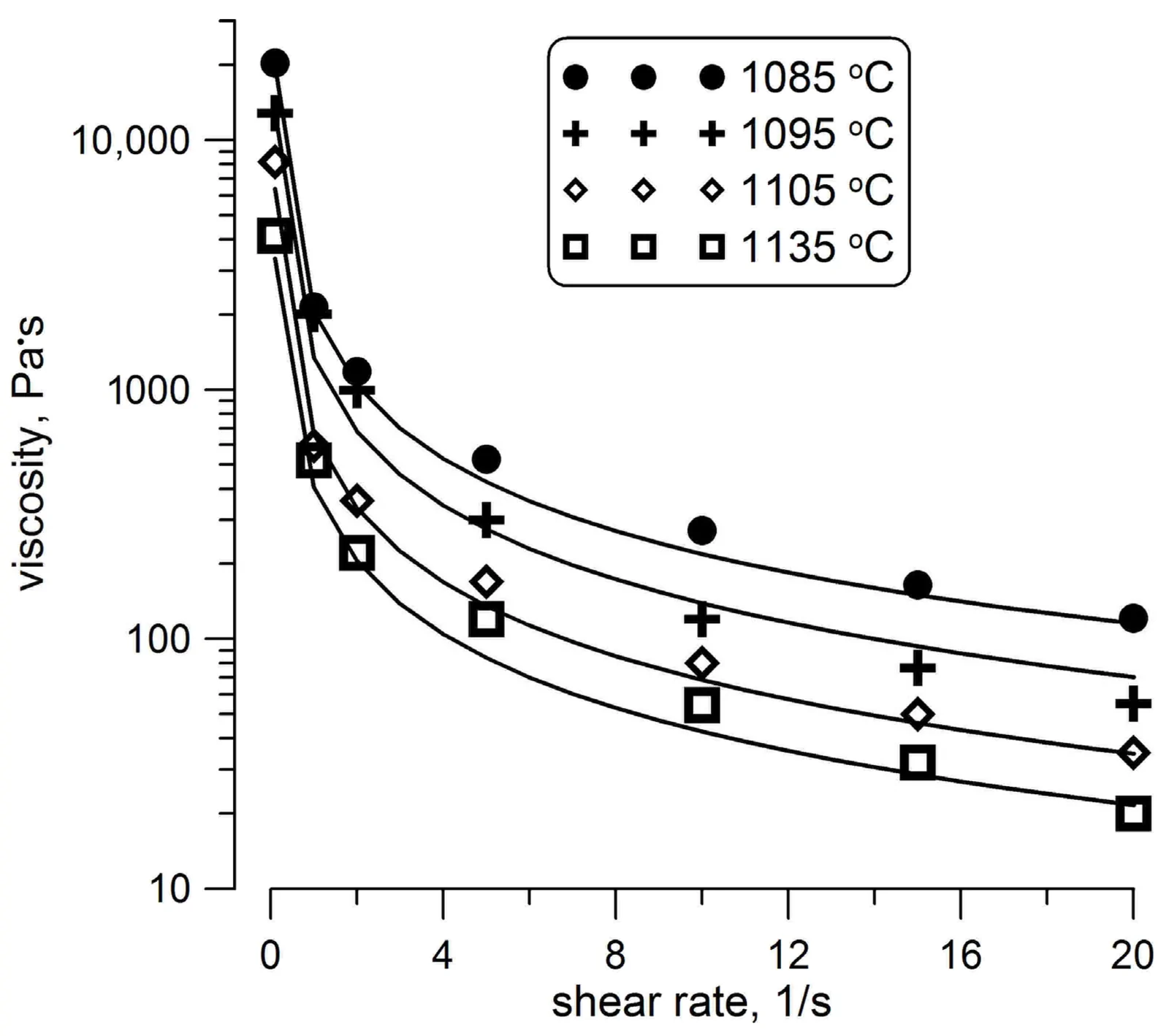
Graph of viscosity versus shear rate calculated using model (5) with points showing measured values of viscosity for CuAl10Fe3Mn2.

Figure 12 shows graphs of rheological parameters of model (5) as a function of temperature. Figure 13 shows the graph of viscosity as a function of shear rate, calculated using model (5) with marked measurement points. This plot shows the accuracy of the approximation using this model. Graphs and approximation were made using the Grapher 7 software (ver. 7.0.1870, Golden Softwae Inc. Golden, CO, USA). This software has an implemented numerical optimisation method that allows minimising of the difference between the measured values and the values calculated by the selected mathematical model whose parameters are estimated. A useful optimisation function is the option to impose constraints on the search space, which makes it possible to search for a solution of physical nature in the case of optimisation of non-unimodal functions.

The approximation of the parameters of model (5) made it possible to determine two characteristic rheological quantities, namely viscosity for infinitely low shear rate and for infinitely high shear rate. The difference of these values makes it possible to determine the sensitivity of viscosity to changes in shear rate for non-Newtonian fluids.

The last model developed in the work is a function of both temperature and shear strain rate (6) [33,34]. This model, among several parameters, has an internal variable in the form of a fraction of the solid phase, modelling the progress of the crystallisation process. However, this progress was described by the Scheil equation used in the modelling of non-equilibrium crystallisation conditions (7). Models of this type cannot always be used in simulation programs, but some packages provide the source codes, making it possible to program such models.

(6) $$\eta =A\cdot \exp\left(B{\cdot f}_{s}\right)\cdot {\dot{\gamma }}^{m}$$

(7) $$f_{s}=1-{\left(\frac{T_{f}-T}{T_{f}-T_{L}}\right)}^{\frac{1}{k-1}}$$

(8) $$k=\frac{C_{S}}{C_{L}}$$

where

- $\dot{\gamma }$—shear rate;
- *A*, *B*, *m*—rheological parameters;
- *fs*—solid phase fraction;
- *T_L_*—liquidus temperature;
- *T_f_*—freezing point of the pure base component;
- *k*—partition coefficient;
- *C_S_*, *C_L_*—element content in the solid and liquid phase.

Equation (6) is a transformation of the Ostwald-de-Waele model *η* = *K*$\dot{\gamma }$*^n^*^−1^, where *K* = *A*exp(*Bf_s_*) and *n*−1 = *m*. In this case, for shear-thinning behaviour, the value of *m* should fall within the range −1 < *m* < 0. A value of *m* = 0 indicates a Newtonian fluid; viscosity is constant and independent of the shear rate, and the parameter *K* becomes the dynamic viscosity. A value of *m* > 0 indicates a dilatant fluid exhibiting shear-thickening behaviour, where viscosity increases with the shear rate. A value of −1 < *m* < 0 indicates a fluid exhibiting shear-thinning behaviour, where viscosity decreases as the shear rate increases. Some researchers have observed anomalous behaviour in semi-solid alloys where *m* < −1 [17]. Such a situation may be caused by structural factors.

**Figure 14:**
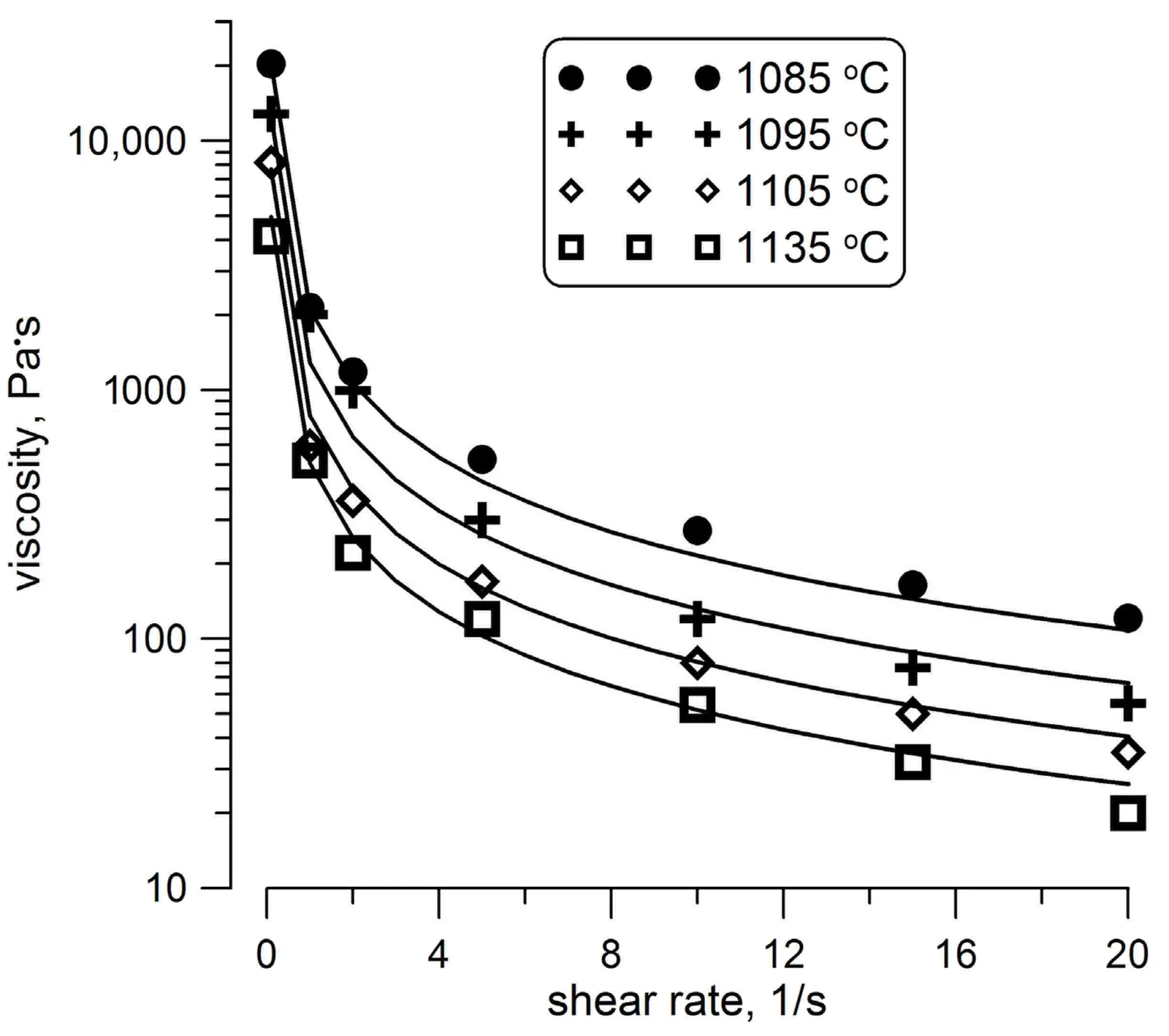
Graph of viscosity versus shear rate calculated using model (6) with points showing measured values of viscosity for CuAl10Fe3Mn2.

Table 4 presents the values of rheological parameters for the dependence of viscosity on shear rate, which was measured. Figure 14 shows the graph of viscosity as a function of shear rate, calculated using model (6) with marked measurement points. This plot shows the accuracy of the approximation using this model. The fitting of model (6) was also assessed by the coefficient of determination *R*^2^, which equals in the case of CuAl10Fe3Mn2 alloy 0.99996, 0.99667, 0.99914 and 0.99948 for temperatures of 1085, 1095, 1105, and 1135 °C, respectively.

**Figure 15:**
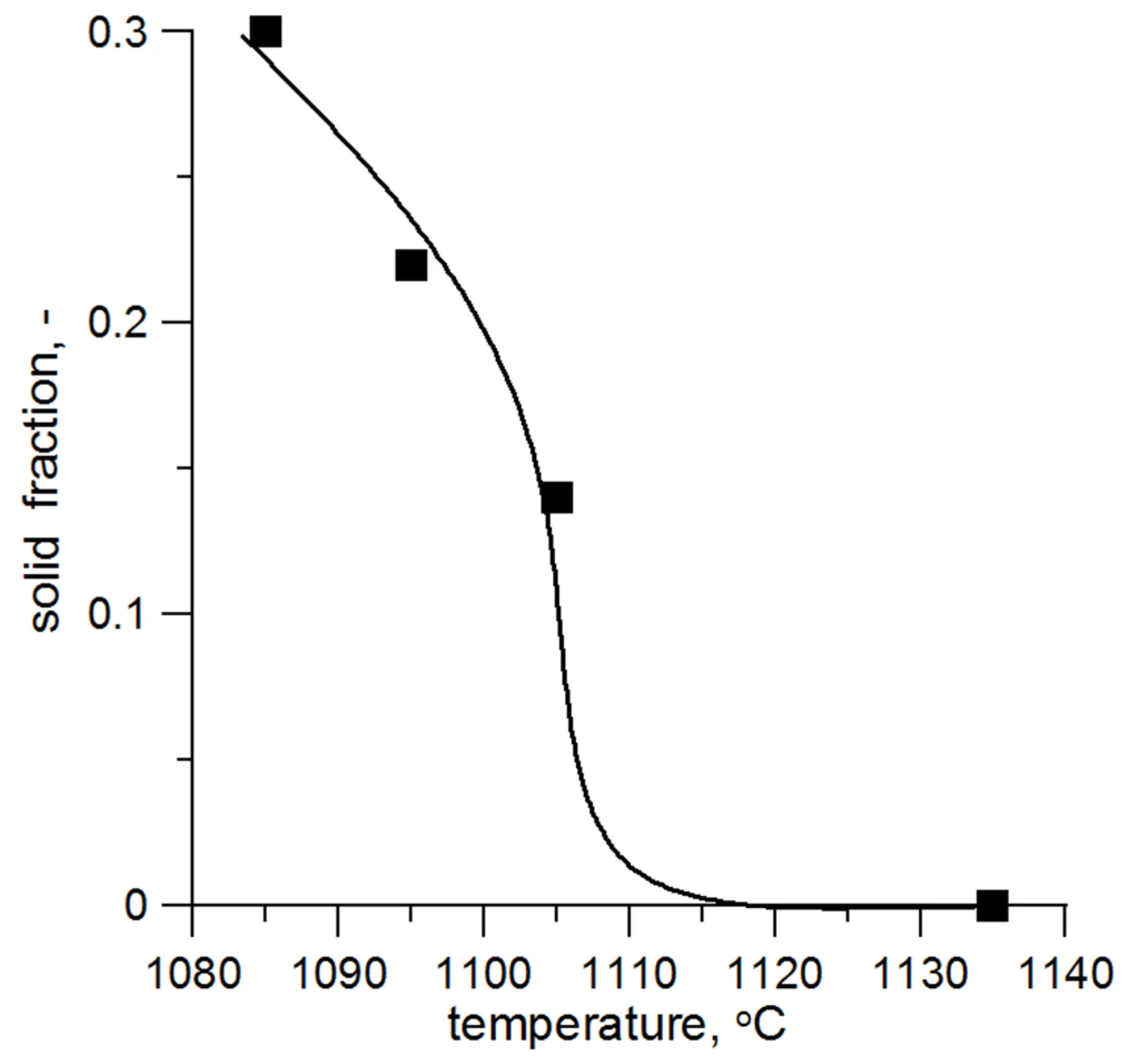
Solid fraction curve versus temperature for CuAl10Fe3Mn2 copper alloy, internal parameter of model (6).

In the rheological model (6), there is an internal parameter (solid fraction), describing the progress of the solidification process. The value of this parameter indicates the amount of the solid fraction in the mushy zone. The course of solidification normally results from the kinetics of the phase transformation. Thixoforming processes take place in non-isothermal conditions, then sometimes the Scheil Equation (7) is used. Especially, it is possible for a binarysystem. In the case of CuAl10Fe3Mn2 the solid fraction curve was determined using thermodynamic databases in the ProCAST software. In Figure 15 the solid fraction curve is shown for theCuAl10Fe3Mn2 alloy.

## 6. Viscosity Sensitivity Analysis

Phenomena of shear tinning, melting and thixotropy of alloys simultaneously cause decrease in alloy viscosity, and the individual influence of these three phenomena was determined using sensitivity analysis for the CuAl10Fe3Mn2 alloy. Rheological models allow the determination of derivatives of viscosity with respect to temperature, shear rate and time (9).

(9) $${\left|\frac{\partial \eta }{\partial T}\right|}_{{\dot{\gamma }}_{\mathrm{const}}}{\left|\frac{\partial \eta }{\partial t}\right|}_{{\dot{\gamma }}_{\mathrm{const}},T_{\mathrm{const}}}{\left|\frac{\partial \eta }{\partial \dot{\gamma }}\right|}_{T_{\mathrm{const}}}$$

**Figure 16:**
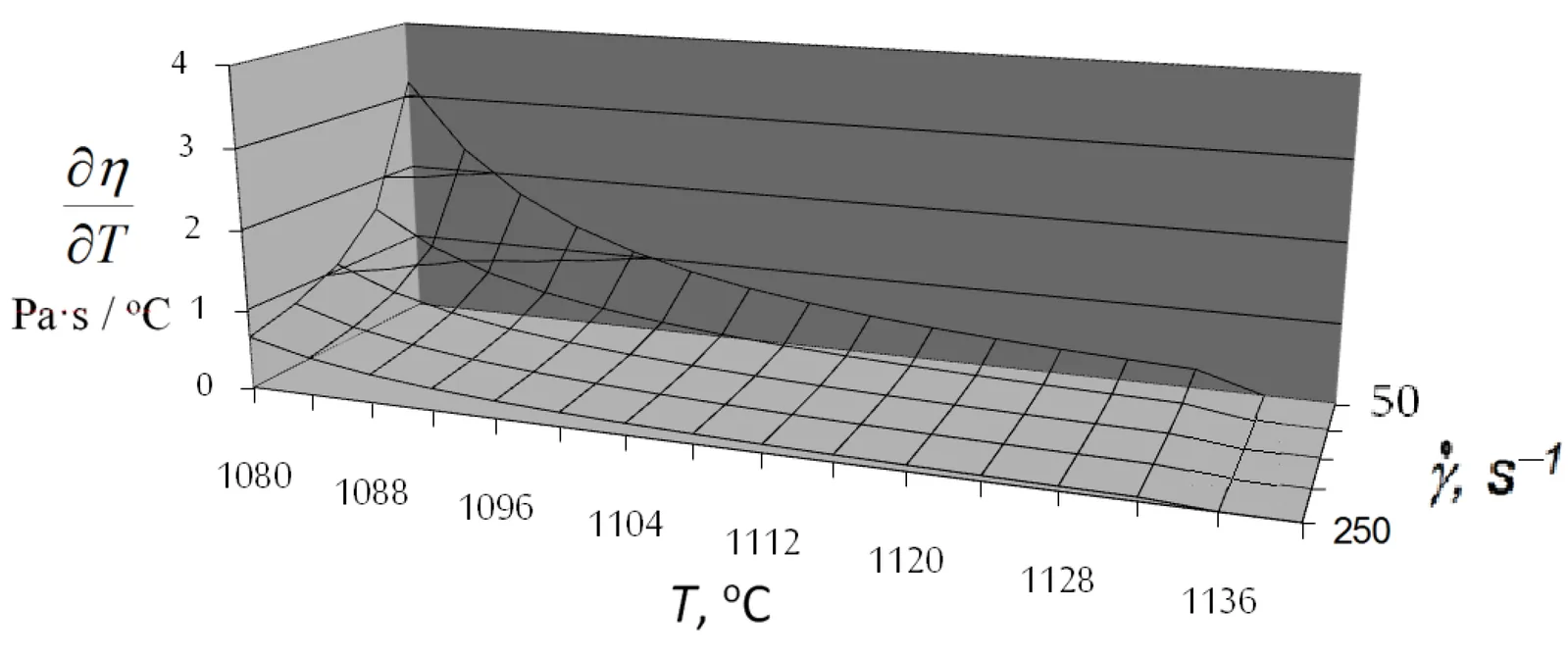
Sensitivity of viscosity model (6) to temperature.

**Figure 17:**
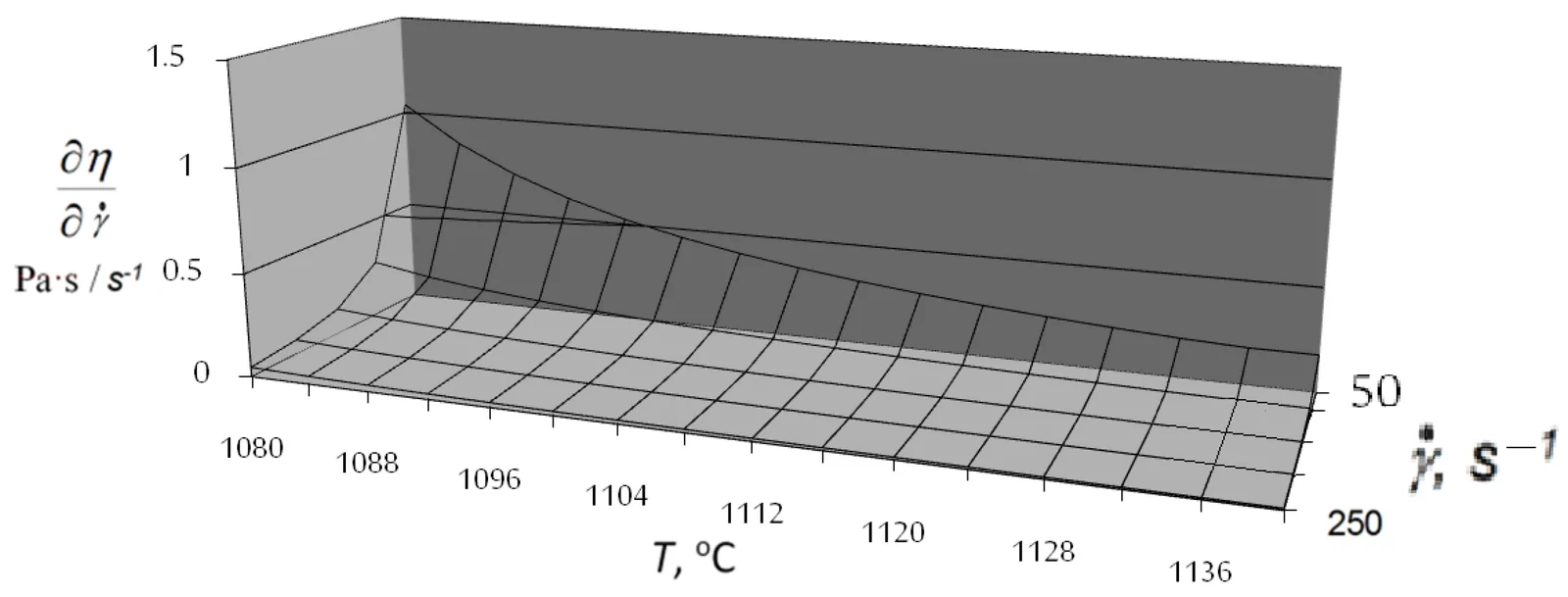
Sensitivity of viscosity model (6) to shear rate.

**Figure 18:**
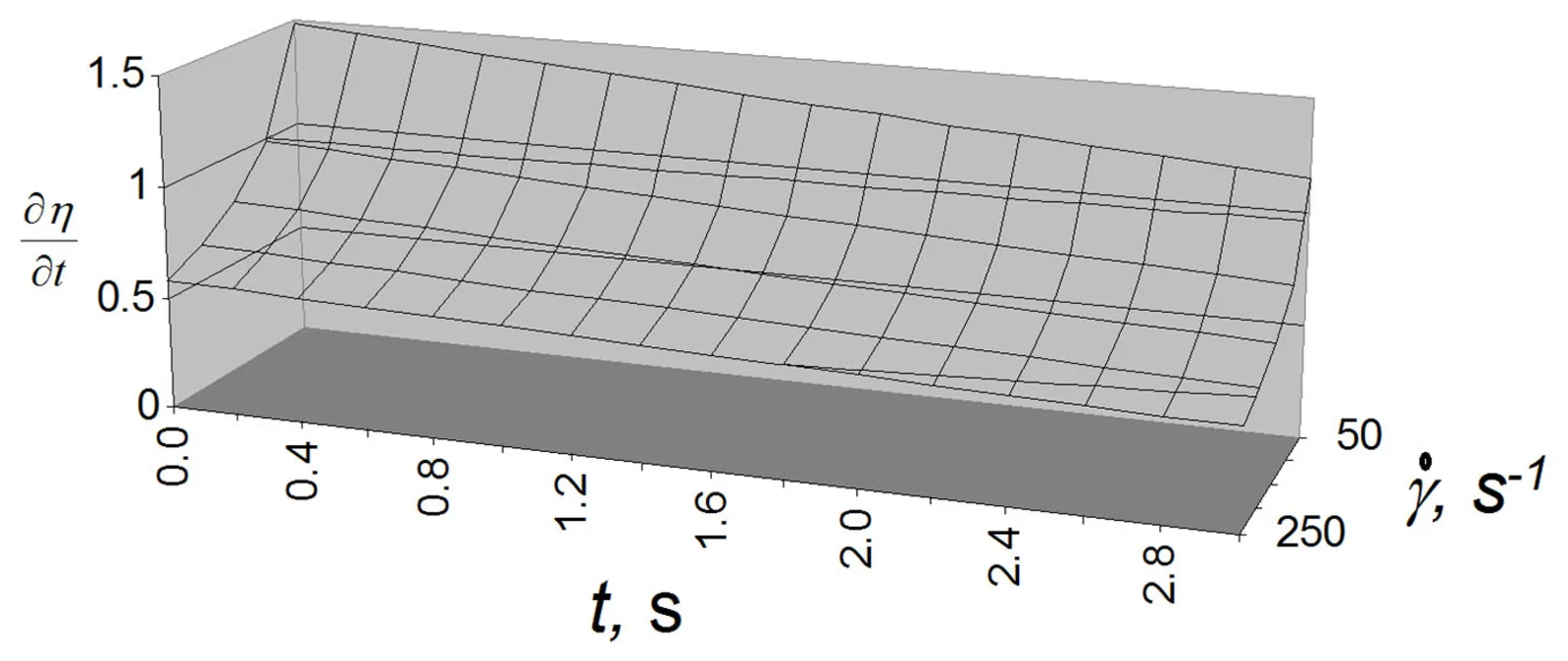
Sensitivity of viscosity model (6) to time.

Figure 16 shows the values of the viscosity derivative with respect to temperature, Figure 17 shows the values of the viscosity derivative with respect to shear rate and Figure 18 the values of the viscosity derivative with respect to time.

## 7. Discussion

The experimental work clearly shows a significant increase in viscosity values with decrease in temperature. This phenomenon is due to the increase in the fraction solid phase in the alloy, as the solidification process progresses. Figure 1 shows that values of viscosity change in a relatively small range of temperature. For example, in the CuSn10 alloy, viscosity decreases about one hundred times in a range of about 100 °C, whilst, for theCuAl10Fe3Mn2 alloy, the decrease in viscosity is much smaller, around ten times. Figure 4 and Figure 5 point to one further relationship, i.e., that there are different ranges of viscosity for different values of shear rate and, in general, lower values of viscosity are observed for higher shear rates.

The last observation was the reason for extending the research to include an analysis of the influence of shear rate on viscosity. Figure 4 and Figure 5 show curves of viscosity versus shear rate for different values of temperatures, with measurements carried out for constant values of temperature and, between these measurements, the alloys were simultaneously sheared at a constant rate of 5 s^−1^ and then the temperature changed to the next value.

Measurements to identify and confirm the phenomenon of thixotropy were carried out. For this, an analysis of viscosity changes during the time of shearing of the samples was carried out for constant temperatures and shear rates. Measurement results are shown in Figure 6 and Figure 7. The CuSn10 alloy was tested at 955 °C and the CuAl10Fe3Mn5 alloy at 1095 °C. The influence of time on viscosity was analysed for different values of shear rates of 2, 10, 20, 25 and 35 s^−1^ and, in each case, the changes in viscosity over time were observed.

Within the confines of this work, identification of the yield stress in the semi-solid state was also carried out. Results for the CuAl10Fe3Mn2 alloy are shown in Figure 8 and Figure 9, and each figure relates to a different temperature. Measurements at 1080 °C are shown in Figure 8 and at 1090 °C in Figure 9. These results show the occurrence of yield stress which is dependent on the length of rest time between successive stages of shearing.

Additionally, mathematical modelling of viscosity was performed, using different rheological models (4), (5) and (6) fitted to precisely describe the behaviour of viscosity. For each alloy, model parameters were estimated using optimisation methods. Figure 10 and Figure 12 show values of rheological parameters versus shear rate and temperature, respectively, and Figure 11, Figure 13 and Figure 14 a comparison of calculated viscosity values with measured values, with each case showing the high precision of the models.

The observed discrepancy in approximating of the measurement results using model (6) may result from two reasons. The first is an imprecise determination of the relationship between the solid phase fraction *fs* and temperature. The second reason is the simplified structure of model (6), which does not account for the variation inparameters A, B, or n as a function of the solid phase fraction—as is in the case, for example, in study [44]. Therefore, our future work will involve precise calorimetric investigations using the DSC technique to improve the accuracy of the *fs* = f(*T*) relationship and the application of a more sophisticated model than the one used in the present study.

Finally, the sensitivity of viscosity to all three parameters, whose influence was investigated, was analysed, and the results are shown in Figure 16, Figure 17 and Figure 18. Comparison of the graphs in Figure 16 and Figure 17 shows that the influence of temperature is stronger than shear rate; this conclusion is drawn from results from higher values of viscosity derivatives with respect to temperature.

## 8. Conclusions

As part of this publication, several pieces of work were carried out toidentify the rheological properties of the selected copper alloys in the liquid and semi-solid states in terms of potential shaping processes. First, the alloys were selected in terms of semi-solid stability from among the available bronzes and brasses. The alloys that successfully meet the requirements of such processes turned out to be the CuSn10 andCuAl10Fe3Mn2 copper alloys.

Subsequently, a high-temperature rheometer was adapted to measure the viscosity of the copper alloys in the semi-solid state. This rheometer was originally designed to measure the viscosity of liquid slags and metals, and the adaptation consisted in designing appropriate tools and measurement procedures.

The next stage of the work was the measurement of the viscosity of the CuSn10 and CuAl10Fe3Mn2 alloys as a function of temperature and shear rate. These measurements allowed us to classify these alloys as non-Newtonian fluids in which the phenomenon of shear thinning occurs. Each recorded curve was characterised by a decrease in viscosity with increasing shear rate. Viscosity variation as a function of shear rate means that the CuSn10 and CuAl10Fe3Mn2 alloys in the semi-solid state are non-Newtonian fluids. On the other hand, the non-linear decrease in viscosity as a function of shear rate allows the alloys to be classified as pseudoplastic liquids. This means that the phenomenon of shear thinning occurs in them. This property is very important from the point of view of thixoforming processes, which use the liquefaction of metal alloys caused by high shaping speeds. In addition, experimental work allowed us to determine the sensitivity of viscosity to temperature and shear rate. Due to the phase transformation that the alloys are undergoing during the progress of crystallisation, a much greater impact of temperature changes on viscosity was noticed than in the case of changes in shear rate.

Due to the potential use of the material characteristics obtained in laboratory tests in numerical simulations of shaping processes, several rheological models of the tested alloys were developed. Thanks to extensive modelling work, viscosity models depending on temperature, shear rate and both variables at the same time were created.

A secondary result of the research was the estimation of the temperature ranges of thixoforming of analysed alloys. Thixocasting processes are carried out on modified high-pressure casting machines. Due to the much lower pressure of the pistons in high-pressure machines compared to forging presses, forming processes should be carried out at higher temperatures with lower viscosity values. In the case of the tested CuAl10Fe3Mn2 alloy, for example, the range of such temperatures is between 1080 and 1100 °C.

## Figures and Tables

**Table 1:** Mechanical properties and temperature ranges of mushy zones for selected bronzes.

Bronze Grade	Tensile Strength, MPa	Yield Stress, MPa	Elongation, %	ΔT_sol-liq_, °C
Tin bronzes				
CuSn6	410	270	30	144
CuSn10	300	170	12	157
CuSn12	280	150	5	155
Silicon bronze				
CuSi3	350	120	40	70
Aluminium bronzes				
CuAl8Fe3	500	200	24	30
CuAl5	390	150	23	8
CuAl10Ni5Fe4	700	330	15	14
CuAl9Mn2	550	200	14	12
CuAl10Fe3Mn2	600	330	8	60

**Table 2:** Values of parameters of rheological model (4).

$\dot{\gamma }$, s^−1^	*A*, Pa·s	*B*, K	*R* ^2^
CuSn10			
0.1	3.90 × 10^−14^	37,086.3	0.996
2	2.62 × 10^−18^	44,000.0	0.999
10	6.04 × 10^−20^	45,280.2	0.999
20	6.94 × 10^−21^	46,517.7	0.999
CuAl10Fe3Mn2			
0.1	1.70 × 10^−13^	42,613.8	0.973
2	1.00 × 10^−14^	42,657.4	0.882
10	1.00 × 10^−15^	43,382.6	0.839
20	1.00 × 10^−16^	45,011.9	0.872

**Table 3:** Values of parameters of rheological model (5).

T, °C	*η*_0_, Pa · s	*η*_∞_, Pa · s	*λ*, s	*a*, -	*n*, -	*R* ^2^
CuSn10						
925	40,000	10	38	12	0.01	0.980
955	10,000	0.15	35	11	0.012	0.998
985	2000	0.01	30	10	0.015	0.957
1020	100	0.001	7	5	0.02	0.979
CuAl10Fe3Mn2						
1085	75,000	10	38	12	0.01	0.999
1095	45,000	0.4	35	11	0.013	0.996
1105	17,000	0.1	27	10	0.015	0.999
1135	3500	0.01	9	8	0.02	0.999

**Table 4:** Values of parameters of rheological model (6).

*A*, Pa·s^m+1^	*B*, -	*m*, -
CuSn10		
13.76	9.79	−0.99
CuAl10Fe3Mn2		
48,172	4.92	−0.99

## Data Availability

The original contributions presented in this study are included in the article. Further inquiries can be directed to the corresponding author.
